# Discrepancies in regional lung cancer multidisciplinary team decisions can be reduced through national consensus meetings

**DOI:** 10.2340/1651-226X.2025.43314

**Published:** 2025-06-16

**Authors:** Anja Gouliaev, Weronika Maria Szejniuk, Joan Fledelius, Hans Henrik Torp Madsen, Rene Horsleben Petersen, Torben Riis Rasmussen

**Affiliations:** a Department of Respiratory Diseases and Allergy, Aarhus University Hospital, Aarhus, Denmark; b Department of Clinical Medicine, Aarhus University, Aarhus, Denmark; c Department of Oncology & Clinical Cancer Research Center, Aalborg University Hospital, Aalborg, Denmark; d Department of Clinical Medicine, Aalborg University, Aalborg, Denmark; e Department of Nuclear Medicine and PET Center, Aarhus University Hospital, Aarhus, Denmark; f Department of Radiology, Aarhus University Hospital, Aarhus, Denmark; g Department of Cardiothoracic Surgery, Copenhagen University Hospital, Rigshospitalet, Copenhagen, Denmark; h Department of Clinical Medicine, University of Copenhagen, Copenhagen, Denmark; i The Danish Lung Cancer Group, Aarhus, Denmark

**Keywords:** lung cancer, Scandinavian and Nordic countries, multidisciplinary team conference, tumor board, consensus

## Abstract

**Background:**

Multidisciplinary team (MDT) meetings are a gold standard in lung cancer care. A recent study identified discrepancies in staging and treatment recommendations among Danish lung cancer MDTs based on fictitious cases. This short report presents the results from a national lung cancer MDT meeting, which reevaluated these difficult cases.

**Method:**

Fifteen difficult cases were reevaluated by 52 lung cancer specialists from across Denmark, representing oncology, pulmonology, radiology, nuclear medicine, and thoracic surgery. Participants were grouped together with their usual MDT colleagues. Cases were presented in a plenary session, and participants discussed cases staging, treatment intent, and treatment options as they would in a regular MDT with their colleagues. If disagreement between the individual MDT groups occurred, the case was further discussed in plenum. Descriptive statistics were used to assess agreement.

**Results:**

Complete agreement on tumor node metastasis (TNM) staging, treatment intent, and recommended treatment was reached in three cases (20%). Agreement on stage was reached in 10 cases (67%). Discrepancies regarding stage arose from debates regarding multifocal versus synchronous lung cancers, degree of lymph node involvement and the malignancy status of pleural fluid. Differences in treatment recommendations were mainly due to insufficient information about performance status.

**Interpretation:**

Staging and treatment intent discrepancies among Danish lung cancer MDTs were considerably reduced when complex cases were discussed in a national plenary session. However, for difficult lung cancer cases, MDTs recommend different treatment, highlighting the need for a national MDT meeting for a select group of lung cancer patients.

## Introduction

The multidisciplinary team (MDT) approach is regarded as the gold standard in lung cancer care. The MDTs have been shown to enhance the accuracy of patient staging and improve survival outcomes for lung cancer patients [1–4]. Patients with stage III non-small cell lung cancer (NSCLC), representing locally advanced disease, frequently present challenges in accurate staging and in planning optimal treatment [5, 6]. Treatment intent for stage III NSCLC patients can be either curative or palliative depending on rather small differences. A recent study evaluating agreement between lung cancer MDTs across Denmark, revealed notable discrepancies in staging and treatment intent among those patients [7] with MDTs disagreeing in 16 out of 60 (27%) fictitious cases. Due to these unexpected discrepancies in the previous study, a national lung cancer MDT consensus meeting was arranged. In this article, we present results from this meeting, where 15 of these cases were reevaluated by the MDTs and discussed collectively.

## Method

This consensus study builds on a prior lung cancer MDT study involving 60 fictitious cases, which were reviewed during lung cancer MDT meetings at four Danish university hospitals (Copenhagen, Odense, Aarhus and Aalborg). Construction and validation of the cases along with characteristics of the cases have been described in detail previously [7]. From the 60 cases in the previous study, the 15 with the most significant discrepancies in tumor node metastasis (TNM) staging or treatment intent between the centres were chosen for this consensus study.

Lung cancer specialists from across the country, involved in diagnosis and treatment, were invited to participate in the consensus meeting held on the 4^th^ of October 2024. Fifty-two specialists across all healthcare regions in Denmark representing oncology, pulmonology, radiology, nuclear medicine, and thoracic surgery, met in person to discuss the 15 selected cases. The specialists were seated in teams with their regional MDT colleagues. The day commenced with an introduction on the purpose of the meeting as consensus seeking, followed by presentations with updates on treatment modalities, including recent advancements in early-stage NSCLC treatment and neoadjuvant chemo-immune therapy. The fictitious lung cancer cases were presented as they had been actual patients at a typical MDT meeting. Full Computed Tomography (CT) and Positron Emission Tomography (PET CT) scans for each case were available at the meeting, presented by specialists in radiology and nuclear medicine. Each of the five MDT groups from each of the five Danish healthcare regions separately discussed each case with regard to TNM stage version 8, treatment intent, and potential treatment options. Responses were submitted electronically by one representative from each MDT group followed by plenary discussions about the individual groups’ decisions. Two cases, for which there had been full agreement in the prior study, were first presented to test the setup, but were not included in the results. Then the 15 cases for which there had previously been disagreement on either stage or intent of treatment were presented and discussed in groups of five cases over a 90-minute period, followed by a break before the next set of five cases. If the groups reached consensus on the TNM stage and treatment plan during their collective discussion, the next case was presented. In case of disagreement, discussion continued until consensus was achieved or it was concluded that disagreement persisted.

### Statistics

Descriptive statistics were used to describe agreement between lung cancer MDT groups. Statistical analysis was performed in Excel 2021.

## Results

### Agreement

In the previous study, there had been disagreements between the lung cancer MDT centres in all cases presented and discussed in the consensus meeting. However, at the consensus meeting, where CT scans and PECT-CT scans were presented by the same specialist, full agreement on TNM stage classification, treatment intent and recommended therapy were observed for three out of 15 cases. In two of these cases, prior disagreement had been in staging (IA/IB and IA/IIB) and for one case disagreement was on whether to treat a stage IVB with palliative chemotherapy and immunotherapy or to offer best supportive care.

### Staging

Agreement on stage was achieved in 10 out of 15 cases during the consensus meeting. In contrast, the previous study found agreement on stage in only three out of the 15 challenging cases (Table 1). Reasons for disagreement on stage included four cases of disagreement on whether stage III or IV and one case of discussion of multifocal lung cancer versus synchronous lung cancers (Table 2). In two cases, a small amount of pleural fluid was present at the CT scans without prior testing for malignancy, leading to disagreements on whether it should be stage IIIA or IVA. Full agreement on TNM classification was reached in seven cases.

**Table 1:** Agreement between MDTs.

Agreement between MDT teams	Consensus MDT	Prior MDT study*
On TNM Stage, treatment intent and treatment recommendation	3/15 (20%)	0
On Stage I+II, Stage III or Stage IV	12/15 (80%)	3/15 (20%)
Curability yes/no	8/15 (53%)	0
Treatment recommendation	5/15 (33%)	0

* Results modified from Rasmussen et al. [7]. MDT: multidisciplinary team.

**Table 2:** Disagreement on TNM stage or recommended treatment.

Proposed TNM stage	Reason for disagreement
IA or IVA	Synchronous or multifocal lung cancer. See Figure 1A
IIIA or IIIB	Whether EBUS was required to determine if the patient had N3 disease
IIIA or IIIB	N1 or N2 disease. See Figure 1B.
IIIA or IVA	Malignant pleural fluid? See Figure 1C.
IIIA or IVA	Malignant pleural fluid?
Treatment intent	Disagreement
Curative	CRT or neoadjuvant immunotherapy and chemotherapy prior to surgery.
Curative	CRT alone or in combination with immunotherapy
Curative or palliative	Synchronous or multifocal lung cancer
Curative or palliative	ECOG PS not in presentation, determines if treatment is with curative or palliative intent
Curative or palliative	ECOG PS not in presentation, determines if treatment is with curative or palliative intent
Curative or palliative	ECOG PS not in presentation, determines if treatment is with curative or palliative intent
Palliative	Immunotherapy alone or in combination with radiation

CRT: chemoradiation therapy; ECOG PS: Easter cooperative oncology group performance status; EBUS: Endobronchial Ultrasound.

**Figure 1:**
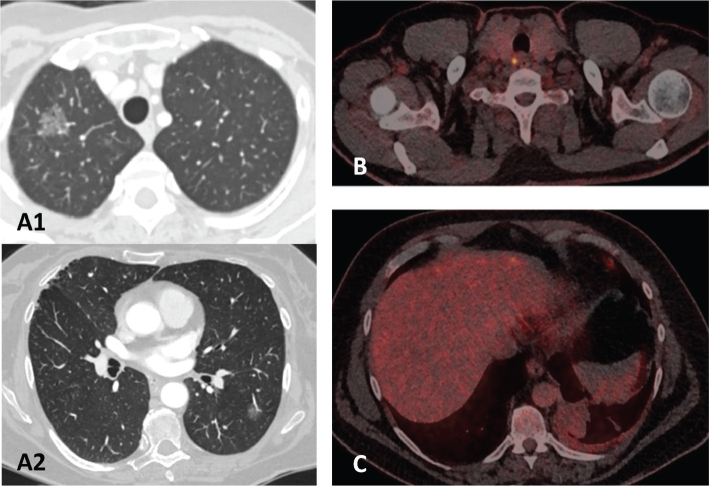
Examples of case with disagreements. (A1+A2) Synchronous or multifocal lung tumours. (B) Possible malignant lymph node in station 1R. (C) Possible malignant pleural fluid.

### Treatment

In eight of the 15 cases, the MDT groups reached agreement on whether treatment could be offered with curative or palliative intent (Table 1). In five cases, there were full agreement on type of treatment, whereas this was only true for one case in the prior MDT study [7]. A list of the disagreement can be found in Table 2. In three cases, consensus on treatment intent could not be reached, as Eastern Cooperative Oncology Group performance status (ECOG PS) was not present in case presentation and was considered essential for some of the MDT groups.

## Discussion

This article reports results from the first national lung cancer MDT consensus meeting in Denmark. Compared to the discrepancies previously presented where cases were discussed independently at four regional MDT meetings [7], we found a higher level of agreement on stage and treatment intent when the same cases were discussed collectively. Despite the MDTs working in groups along their regular colleagues, all cases were presented by the same radiologist and nuclear medicine specialist, which may have contributed to a higher agreement on TNM classification and stage between the MDTs compared to the previous study. Notably, since the previous publication, new treatment modalities have been approved for treatment of locally advanced NSCLC [8]. In line with results from other studies, in four out of five cases disagreement concerned stage III [6]. Stage III NSCLC patients represent a highly heterogeneous group with disease presentation ranging from apparently resectable tumours to unresectable tumours with extensive nodal involvement. In four out of the 15 cases discussed, the MDTs still disagreed on treatment intent, mostly due to missing patient information on ECOG PS. ECOG PS is important, as NSCLC patients with ECOG PS higher than one (indicating lower physical condition), are rarely referred to treatment with curative intent and patients with ECOG PS 3 or 4 are generally prescribed best supportive care. If ECOG PS is missing at MDT, the oncologist will evaluate the patient at the following consultation, in order to recommend a treatment plan. If the case presentations had included information on ECOG PS, the disagreements regarding these cases might have been eliminated. In certain MDT centres, neoadjuvant immunotherapy and chemotherapy prior to surgical resection, are now available, while others have yet to implement these treatments, which may partly have contributed to differences regarding proposed treatment.

## Conclusion

Disagreement of stage and treatment intent between lung cancer MDTs in Denmark, were fewer when complex lung cancer cases were discussed in plenary following joint CT and PET-CT presentations. For a select group of lung cancer patients, the establishment of a national MDT meeting could potentially expand their treatment options and influence decision-making.

## Data Availability

The data collected for this study can be made available to others by contacting the corresponding author.
